# Left ventricular assist devices promote changes in the expression levels of platelet microRNAs

**DOI:** 10.3389/fcvm.2023.1178556

**Published:** 2023-06-15

**Authors:** Maria Lombardi, Marta Bonora, Luca Baldetti, Marina Pieri, Anna Mara Scandroglio, Giovanni Landoni, Alberto Zangrillo, Chiara Foglieni, Filippo Consolo

**Affiliations:** ^1^ Cardiovascular Research Center, IRCCS San Raffaele Scientific Institute, Milano, Italy; ^2^ Università Vita Salute San Raffaele, Milano, Italy; ^3^ Cardiac Intensive Care Unit, IRCCS San Raffaele Scientific Institute, Milano, Italy; ^4^ Department of Anesthesia and Intensive Care, IRCCS San Raffaele Scientific Institute, Milano, Italy

**Keywords:** platelets, microRNA, left ventricular assist device, hemocompatibility, heart failure

## Abstract

**Introduction:**

MicroRNAs (miRs) emerged as promising diagnostic and therapeutic biomarkers in cardiovascular diseases. The potential clinical utility of platelet miRs in the setting of left ventricular assist device (LVAD) support is unexplored.

**Methods:**

We prospectively measured **t**he expression levels of 12 platelet miRs involved in platelet activation, coagulation, and cardiovascular diseases in LVAD patients by quantitative real-time polymerase chain reaction. Data were longitudinally measured before LVAD implant and after 1, 6, and 12 months of LVAD support, and compared with those measured in healthy volunteers (controls). *In silico* analysis was also performed to identify pathways targeted by differentially expressed miRs.

**Results:**

Data from 15 consecutive patients and 5 controls were analyzed. Pre-implant expression levels of platelet miR-126, miR-374b, miR-223, and miR-320a were significantly different in patients vs. controls. The expression levels of platelet miR-25, miR-144, miR-320, and miR-451a changed significantly over the course of LVAD support; *in silico* analysis revealed that these miRs are implicated in both cardiac- and coagulation-associated pathways. Furthermore, the patients who suffered from bleeding (*n* = 5, 33%) had significantly higher pre-implant expression levels of platelet miR-151a and miR-454 with respect to the patients who did not. The same miRs were also differentially expressed in bleeders following LVAD implantation early before the clinical manifestation of the events.

**Discussion:**

This study provides a proof-of-concept evidence of significant modulation of platelet miRs expression driven by LVADs. The possible existence of a platelet miRs signature predictive of the development of bleeding events warrants further validation studies.

## 1. Introduction

Left ventricular assist device (LVAD) therapy improves the functional status and quality of life of patients with advanced heart failure (HF), and long-term survival with modern LVADs continues to increase (1–3). However, hemocompatibility-related adverse events (HRAEs), i.e., bleeding and thromboembolic complications, are frequent in LVAD patients (2, 3).

HRAEs are the result of a progressive change in the coagulation profile of the patients toward a pro-thrombotic/pro-hemorrhagic state driven by different synergic contributory mechanisms (4–14). Nevertheless, the lack of association between HRAEs and standard coagulation parameters highlights a critical gap in their diagnostic and therapeutic pathway. In this scenario, standardized and reliable criteria to stratify LVAD patients according to thrombotic or hemorrhagic risk are missing, and to define a patient-tailored strategy to prevent HRAEs remains a challenge. In particular, tools and biomarkers that are able to predict the derangement of platelet function toward a pro-thrombotic or a pro-hemorrhagic profile are not available.

This clinical need could be potentially met by circulating microRNAs (miRs), tiny non-coding RNAs with regulatory function on multiple target genes, which recently emerged as disease-associated biomarkers in cardiovascular diseases, including HF (15–17).

To date, the investigation on miRs in patients with LVADs is in its infancy, and studies on plasma or cardiac tissue provided heterogeneous data on their predictive potential and relationship with myocardial or vascular remodeling (18–22).

Previous studies highlighted that platelets possess a miR repertoire and processing machinery related to hypo/hyper-reactivity (23), and that miR target genes could be de-repressed upon platelet activation, affecting platelet function both at gene and protein levels in individually variable manner (24). To date, the relation between platelet miRs expression in LVAD patients and occurrence of HRAEs is unexplored.

On this background, we analyzed, for the first time, longitudinal changes in the levels of expression of platelet miRs in the setting of LVAD support. The present study provides a proof-of-concept of the variation of platelet miRs in the setting of durable LVAD support.

## 2. Material and methods

This is a prospective observational study conducted in consecutive patients with advanced HF according to the 2022 American College of Cardiology/American Heart Association/Heart Failure Society of America guideline for the management of heart failure (25) who were listed for LVAD implantation between February 2019 and December 2020 at San Raffaele Scientific Institute (Milano, Italy). The study conforms to the ethical guidelines of the Declaration of Helsinki and was approved by the local IRB (protocol ID: PASVAD, approved: June 2017; ClinicalTrials.gov ID: NCT03255928). All patients signed informed consent to participate in the study.

The expression levels of a panel of 12 platelet miRs (Table 1) were longitudinally measured pre-implant (t0, i.e., between 24 and 48 h before the device implant), and following 1 (t1), 6 (t2), and 12 (t3) months of LVAD support. The miRs expression levels were measured in both platelet-rich plasma (PRP) and platelet-poor plasma (PPP) pellet samples by quantitative real-time polymerase chain reaction (RT-qPCR), as described in the Supplementary Methods, and compared with those measured in *n *= 5 healthy volunteers matched for age and sex (controls). MiR values measured in PRP were normalized against the platelet count in each blood sample.

**Table 1:** List of miRs analyzed and probes for miR determination in RT-qPCR.

miR name	miR ID
hsa-miR-19b-3p	478264_mir
hsa-miR-20b-5p	477804_mir
hsa-miR-25-3p	477994_mir
hsa-miR-126-5p	477888_mir
hsa-miR-144-3p	477913_mir
hsa-miR-151a-3p	477919_mir
hsa-miR-223-3p	477983_mir
hsa-miR-320a	478594_mir
hsa-miR-374b-5p	478389_mir
hsa-miR-382-5p	478078_mir
hsa-miR-451a	478107_mir
hsa-miR-454-3p	478329_mir
hsa-miR-16-5p^a^	477860_mir
hsa-miR-103a-3p^a^	478253 mir

RT-qPCR, quantitative real-time polymerase chain reactions; miR, microRNA.

^a^ Housekeeping miR.

The 12 miRs we analyzed were selected according to previous evidence of their involvement in coagulation, platelet activation, and cardiovascular disease (Supplementary Table S1).

The pre-implant demographics and clinical characteristics of the patients, changes in the clinical data over the course of support, and clinical outcomes, including HRAEs (non-surgical bleeding and thromboembolic complications) were also recorded. The INTERMACS definitions for adverse events were applied (26). Data were retrieved at the longest available follow-up.

### 2.1. Statistical analysis

Categorical data are presented as absolute numbers and percentages and were compared by two-tailed *X^2^* test or Fisher’s exact test. Numerical data are presented as medians and interquartile range (IQR: 25th–75th percentiles). The Shapiro–Wilk normality test was applied to assess the normality of data distribution. Comparison between groups was performed throughout the Student’s *t*-test or the Mann–Whitney *U*-test for normally and non-normally distributed data, respectively.

Analysis of longitudinal data (t0 to t3) was performed with the ANOVA test for correlated samples or the Friedman test for normally and non-normally distributed data, respectively using the linear mixed effects model [restricted maximum likelihood estimation (REML) method]: the model accounts for both fixed effects (different time-points) and random effects (different observations within a patient); Geisser–Greenhouse correction was applied. Tukey *post-hoc* test was applied to evaluate differences between different time-points.

Correlation among clinical and experimental variables was analyzed by Spearman-R test. Simple linear regression or non-linear fit test was applied as opportune. Probability values <0.05 were considered significant. Statistical analysis was performed with GraphPad PRISM v.8.2.0 (GraphPad Software, San Diego, CA, United States).

## 3. Results

### 3.1. Patient characteristics, clinical outcomes, and occurrence of HRAEs

A total of 18 patients were enrolled. One patient (5%) died before LVAD implantation and two (11%) during early post-operative hospital stay and were excluded. Data of the remaining 15 patients were analyzed. The pre-implant demographic and clinical characteristics of the patients are presented in Table 2 together with descriptive characteristics of the controls.

**Table 2:** Pre-implant demographic and clinical characteristics of the patients and descriptive characteristics of the controls.

Variable	Patients (*n *= 15)	Controls (*n *= 5)^a^
Age (years)	64 (63–71)	53 (47–61)
Male sex (*n*, %)	15 (100)	5 (100)
BMI (kg/m^2^)	22 (21–27)	23 (20–26)
Acute cardiac failure (*n*, %)	4 (27)	—
Chronic HF (*n*, %)	11 (73)	—
Ischemic HF etiology (*n*, %)	9 (60)	—
INTERMACS class* (*n*, %)		—
1–2	7 (47)	
3–4	8 (53)	
Diabetes (*n*, %)	3 (20)	0 (0)
ICD device in place at implant (*n*, %)	9 (60)	—
CRT device in place at implant (*n*, %)	5 (33)	—
Temporary MCS (*n*, %)	14 (93)	—
Impella	10 (67%)	
LVAD (*n*, %)		—
HM3	8 (53)	
HVAD	7 (47)	
Intention to treat (*n*, %)		—
BTT	3 (20)	
DT	12 (80)	

BMI, body mass index; HF, heart failure; INTERMACS, interagency registry for mechanically assisted circulatory support [*with temporary circulatory support (TCS) modifier]; ICD, implantable cardioverter-defibrillator; CRT, cardiac resynchronization therapy; MCS, mechanical circulatory support (Intra-aortic balloon pump, Impella, extracorporeal membrane oxygenation); HM3, HeartMate 3™ ventricular assist device (Abbott Laboratories, United States); HVAD, HeartWare™ ventricular assist device (Medtronic Inc., United States); BTT, bridge to transplant; DT, destination therapy.

^a^ No history of hypertension and cardiovascular and renal diseases.

The patients were treated according to guideline-directed medical therapy for HF (25). All patients were alive on LVAD support at the longest follow-up. The median time of follow-up at t1, t2, and t3 was 66 (40–90), 210 (184–223), and 480 (416–512) days, respectively. The changes in the clinical data of the patients over the course of LVAD support are reported in Table 3.

**Table 3:** Changes in patients' clinical data over the course of LVAD support.

Variable	t0 (*n* = 15)	t1 (*n* = 13)	t2 (*n* = 11)	t3 (*n* = 10)	*p*-value
NYHA class (*n*, %)					
I-II	0 (0)	12 (92)	11 (100)	9 (90)	*<0.0001*
III-IV	15 (100)	1 (8)	0 (0)	1 (10)	
Anticoagulation (*n*, %)	15 (100)	13 (100)	11 (100)	9 (90)	0.26
Antiplatelet drugs (*n*, %)	0 (0)	6 (46)	3 (27)	2 (20)	*0.03*
LVEDD (mm)	69 (65–72)	57 (52–64)	60 (58–63)	55 (52–62)	*0.003*
LVEF (%)	18 (10–22)	18 (13–25)	18 (15–24)	18 (15–21)	0.84
RV dysfunction (*n*, %)	5 (33)	5 (38)	3 (27)	5 (50)	0.73
RVEDD (mm)	35 (30–40)	32 (28–36)	35 (27–41)	36 (34–37)	0.06
Valve disease—moderate to severe (*n*, %)					
Mitral regurgitation	14 (93)	4 (30)	5 (45)	4 (40)	0.95
Aortic regurgitation	7 (47)	2 (15)	1 (9)	1 (10)	
Tricuspid regurgitation	10 (67)	3 (23)	3 (23)	1 (10)	
MAP (mmHg)	–	85 (80–87)	83 (80–86)	85 (85–91)	0.42
Hemoglobin (g/dL)	11.4 (10.7–12.5)	11.2 (10.2–12.1)	12.1 (10.6–13.6)	11.4 (10.8–13.4)	0.47
Hematocrit (%)	36 (33–39)	34 (31–36)	37 (33–42)	36 (33–41)	0.10
Platelet count (10^9^/L)	120 (106–152)	195 (142–216)	190 (156–217)	194 (157–234)	*0.06*
LDH (U/L)	394 (312–566)	242 (184–269)	221 (178–267)	248 (198–270)	*0.005*
INR	1.46 (1.36–1.52)	1.95 (1.65–2.21)	1.97 (1.60–2.30)	2.89 (2.13–3.23)	*0.0008*
aPTT (ratio)	1.15 (1.13–1.28)	1.11 (1.05–1.21)	1.09 (1.00–1.19)	1.26 (1.12–1.34)	0.18
D-Dimer (mg/mL)	3.24 (0.98–6.62)	5.64 (2.91–9.22)	1.79 (1.53–1.66)	2.10 (1.53–2.96)	*0.02*
Fibrinogen (mg/dL)	503 (459–651)	378 (342–509)	361 (303–516)	348 (317–487)	*0.04*
AT-III (%)	97 (72–99)	98 (84–104)	104 (79–116)	97 (88–110)	0.22
CRP (mg/L)	36.3 (17.4–60.9)	26.0 (13.8–35.9)	0.96 (0.46–14.8)	1 (0.7–7.2)	*0.0006*
Creatinine (mg/dL)	1.31 (1.13–2.38)	1.20 (0.85–1.70)	1.49 (1.02–1.74)	1.60 (1.09–1.71)	0.50
Heart Failure medications (*n*, %)					
Beta-blockers	11 (73)	10 (77)	7 (64)	7 (70)	0.98
ACEi/ARB	6 (40)	6 (46)	8 (73)	7 (70)	
MRA	6 (46)	4 (31)	8 (73)	4 (40)	
Loop diuretics	13 (87)	13 (100)	9 (82)	9 (90)	
HRAEs (*n*, type)					
Bleeding events	–	3 (epistaxis, anemization, maelena + anemization)	1 (gastro-intestinal)	3 (anemization, 2× intracranial hemorrhage)	–
Thromboembolic events	–	1 (ischemic stroke)	–	–	

LVAD, Left Ventricular Assist Device; t0, pre-implant; *t1*, >1 months of LVAD support; *t2*, >6 months of LVAD support; *t3*, >12 months of LVAD support; NYHA, New York Heart Association; LVEDD, Left Ventricular End Diastolic Diameter; LVEF, Left Ventricular Ejection Fraction; RV, right ventricle; RVEDD, Right Ventricular End Diastolic Diameter; MAP, Mean Arterial Pressure; LDH, lactate dehydrogenase; INR, International Normalized Ratio; aPTT, activated partial thromboplastin time; AT-III, antithrombin III; CRP, C-reactive protein; ACEi, Angiotensin Converting Enzyme inhibitors; ARB, angiotensin receptor blockers; MRA, Mineralocorticoid receptor antagonist; HRAEs, hemocompatibility-related adverse events.

Italic was used for *p*-values <0.05, to highlight statistical significance.

We recorded 8 HRAEs: 7 bleeding events in 5 (33%) patients and 1 thromboembolic event (7% of the patients). The details on the type of bleeding and thromboembolic events are reported in Table 3. Two bleeding events (40%) occurred in patients implanted with the HeartWare HVAD (Medtronic Inc., United States) and three (60%) in those implanted with the HeartMate 3 (HM3; Abbott, United States). The patient who developed a thromboembolic event was implanted with the HVAD. At the time of the event, all patients were treated with oral anticoagulant (warfarin) targeted to an international normalized ratio (INR) of 2–2.5; the patients implanted with the HVAD were also on aspirin (300 mg/die). The median duration of LVAD support at the time of a primary HRAE was 175 (144–331) days.

### 3.2. Pre-implant platelet miRs expression levels

**Figure 1:**
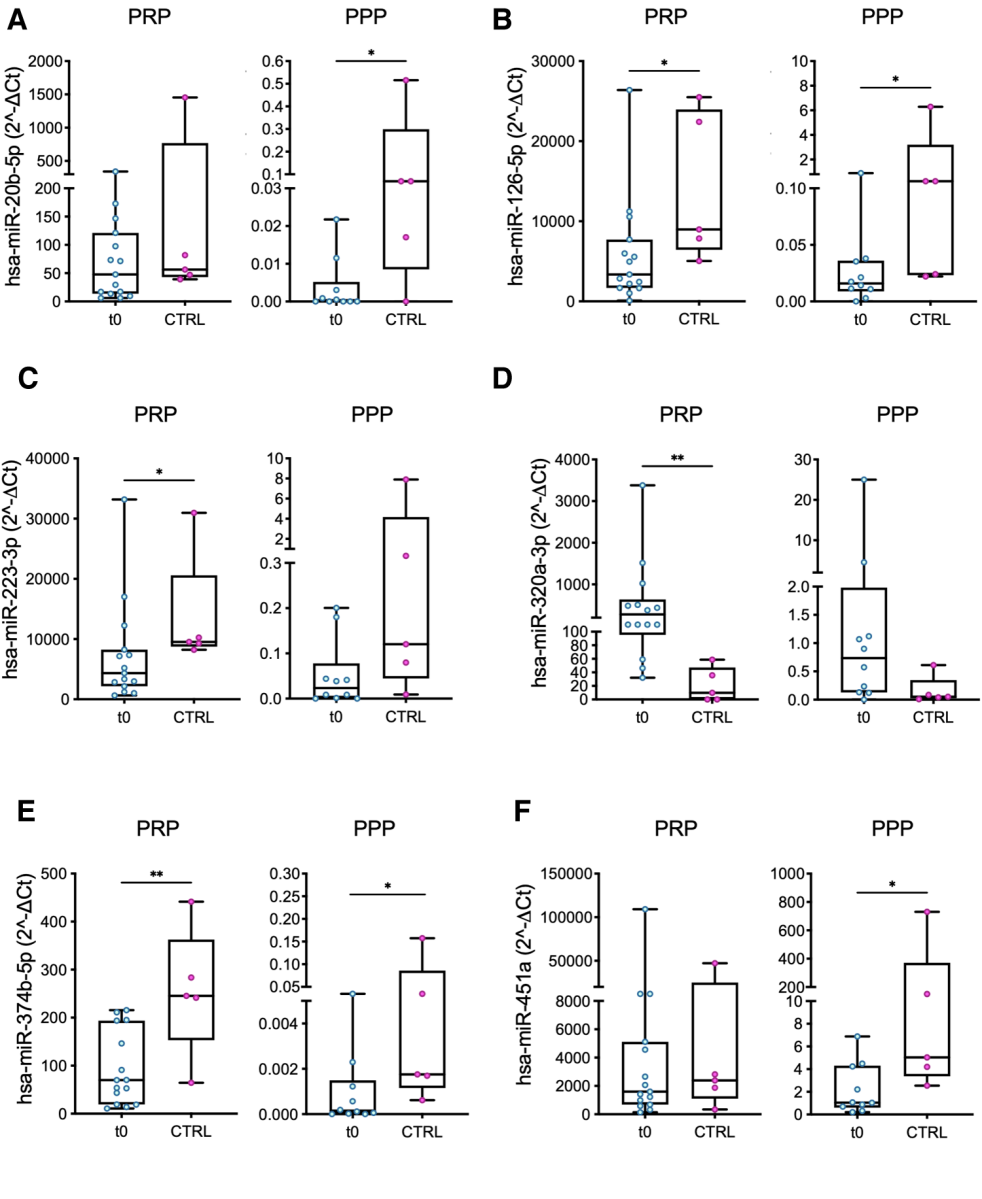
Comparison of miRs expression levels in PRP and PPP between LVAD patients at baseline (t0) and healthy volunteers (controls, CTRL). The miRs with a significant different level of expression between the two groups are shown: (**A**) miR-20b; (**B**) miR-126; (**C**) miR-223; (**D**) miR-320a; (**E**) miR-374b; and (**F**) miR-451a. Values are presented as boxes: dots indicate single values; whisker bars indicate min and max. *p < 0.05; **p < 0.01.

Six out of 12 miRs were differently expressed (DEmiRs; *p *< 0.05) in patients before LVAD implantation vs. controls: (i) miR-126 and miR-374b in both PRP and PPP, (ii) miR-223 and miR-320a in PRP, and (iii) miR-20b and miR-451 in PPP (Figure 1). Moreover, consistent with the PRP pattern, differences in the expression levels of miR-223 and miR-320a in PPP reached borderline significance (*p *= 0.053 and *p *= 0.055, respectively).

In all but one case (miR-320a), the median values of miRs expression level were lower in patients vs*.* controls (Figure 1).

The miRs expression levels were higher in PRP with respect to PPP in all the samples (Figure 1), highlighting the actual expression of miRs by platelets and the existence of a minor—though detectable—miR circulating fraction in PPP potentially generated by activated platelets and carried by lipoproteins of bear by microvesicles (27, 28).

*In silico* analysis revealed that 4 out of 6 DEmiRs, i.e., miR-20b, miR-223, miR-320a, and miR-374b, share a common target gene: the sodium voltage-gated channel alpha subunit 1 (SCN1A). No shared targets were found for miR-126 and miR-451a and the other DEmiRs. (Supplementary File S1).

No significant differences were found between patients and controls for miR-144, miR-151a, miR-19b, miR-25, miR-382, and miR-454 either in PRP or PPP (Supplementary Figure S1).

**Figure 2:**
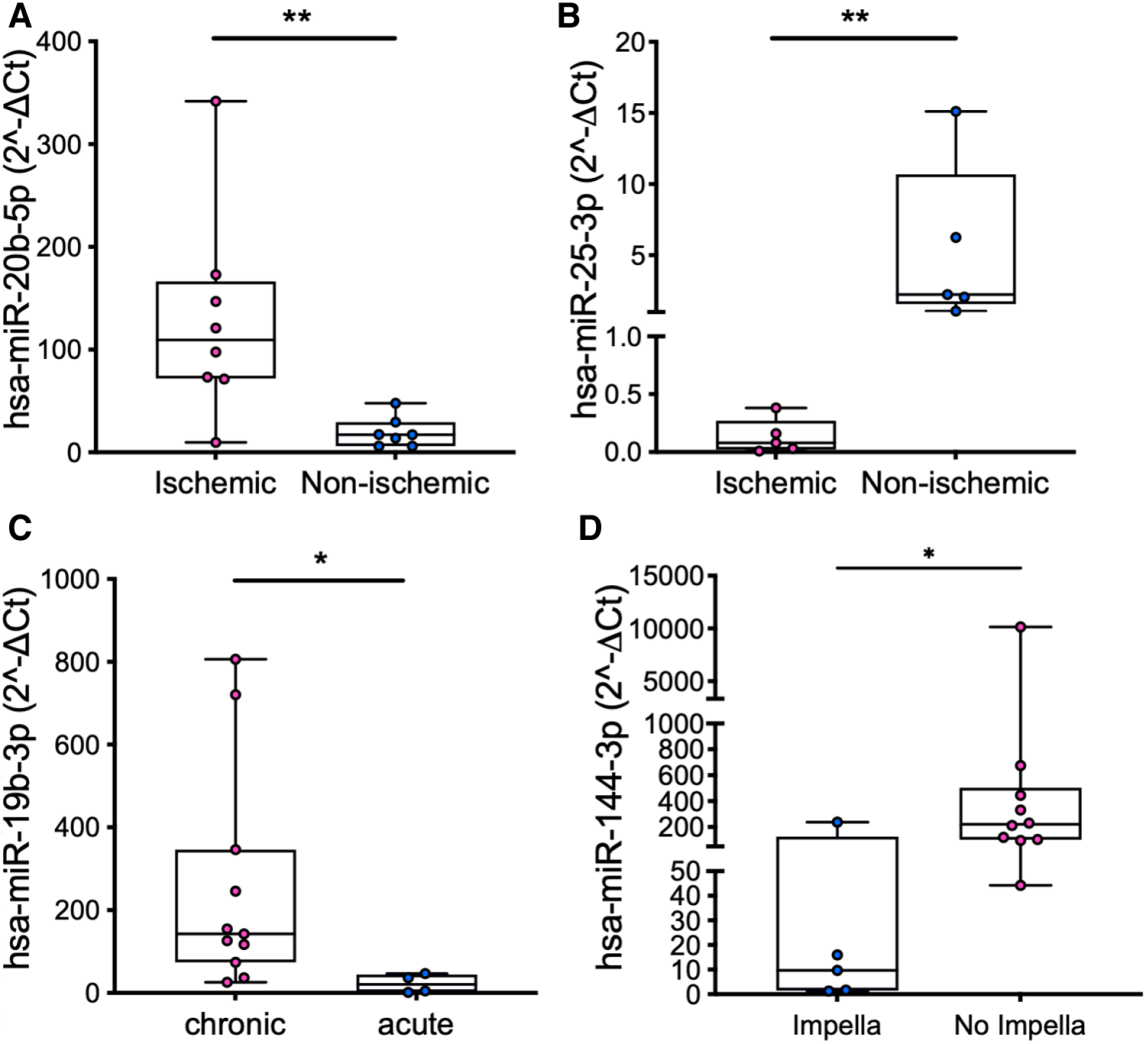
Comparison of the expression levels of miR-20b in PRP (**A**) and miR-25 in PPP (**B**) in patients with ischemic vs. non-ischemic etiology of HF, miR-19b in PRP in patients with chronic vs. acute cardiac failure (**C**), and miR-144 in PRP in patients who were vs. were not treated with an Impella device (**D**). Values are presented as boxes: dots indicate single values; whisker bars indicate min and max. *p < 0.05; **p < 0.01.

Subgroup analysis in the patients revealed significant differences in the miRs expression levels according to either patients’ characteristics (ischemic vs. non-ischemic etiology of HF and chronic vs. acute cardiac failure, Figures 2A–C) or treatment received before LVAD implant (Impella device, Figure 2D).

### 3.3. Changes in platelet miRs expression over the course of LVAD support

**Figure 3:**
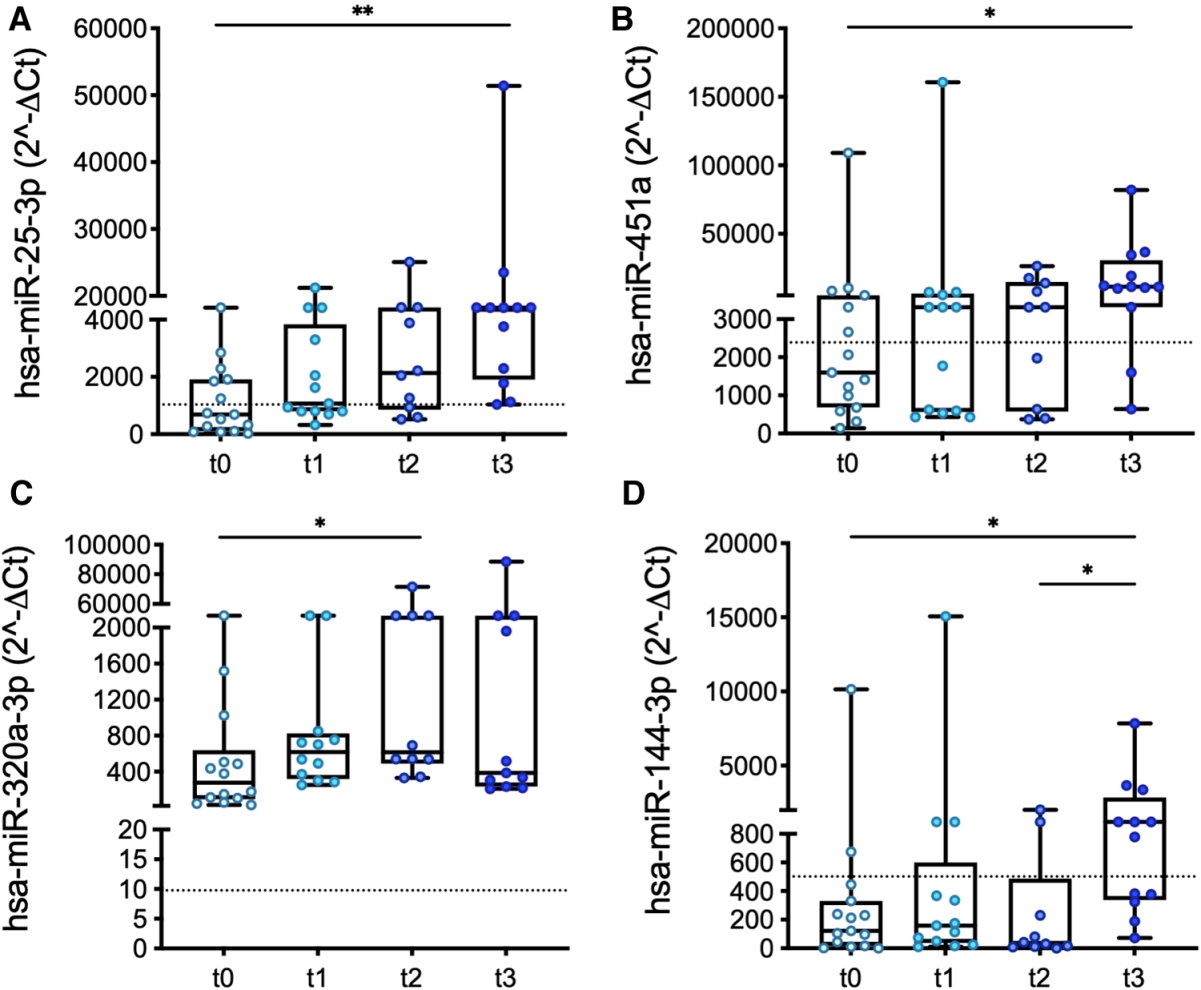
Longitudinal changes in the expression levels of miR-25 (**A**), miR-451a (**B**), miR-320a (**C**), and miR-144 (**D**) measured in PRP over the course of LVAD support. Values are presented as boxes: dots indicate single values; whisker bars indicate min and max. Dashed lines indicate the median value of expression in controls. *p < 0.05.

The expression levels of four miRs measured in PRP changed significantly from t0 to t3: miR-25, miR-451a, miR-320a, and miR-144, (Figure 3). The differences become significant in the long-term (i.e., at t2 or t3).

Interestingly, significant differences (miR-320a; Figure 1) and/or similarities (miR-25, miR-451a, and miR-144; Figure 1 and Supplementary Figure S1) determined at baseline in patients vs. controls were confirmed at the longest follow-up, except for platelet miR-25, whose expression levels in patients vs. controls were comparable at t0 but then progressively increased in the patients (Figure 3A).

**Figure 4:**
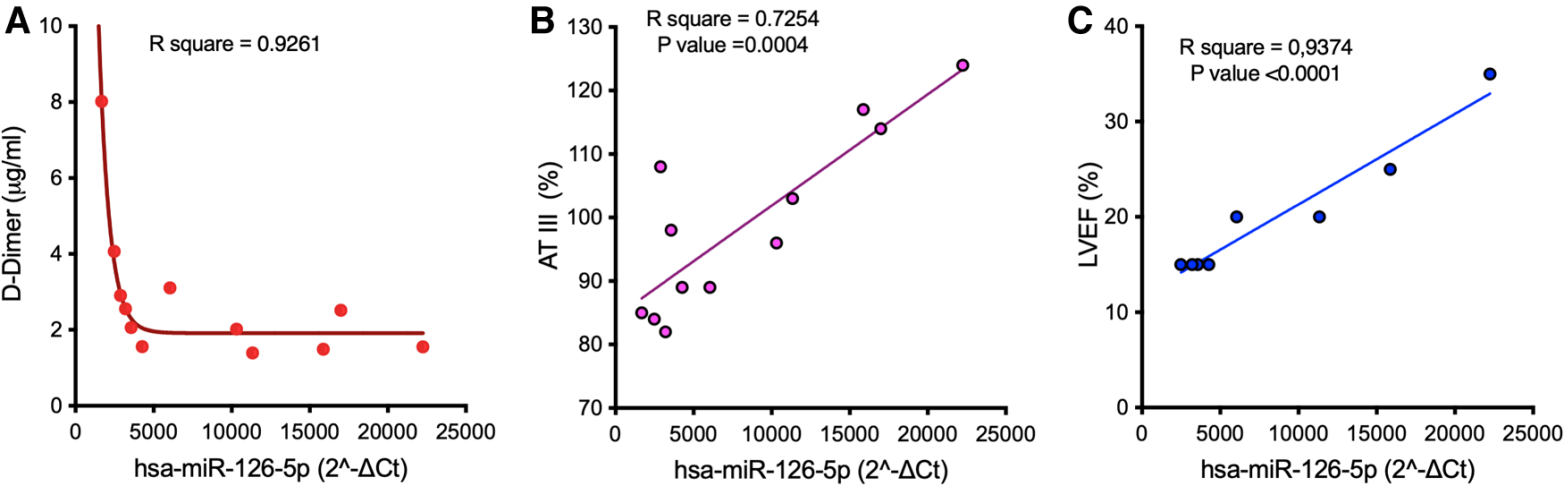
Correlation between the median expression of miR-126 in PRP in samples harvested at t3 from LVAD patients and D-Dimer (**A**) and AT-III (**B**) levels, and metrics of left ventricular function (**C**). AT-III, antithrombin III; LVEF, left ventricular ejection fraction.

The expression level of miR-126 in PRP—but not of other miRs—showed a good correlation at t3 with D-Dimer and AT-III levels (Figures 4A,B) as well as with metrics of left ventricular function (left ventricular ejection fraction, LVEF; Figure 4C).

No further correlations were found between platelet miRs and other clinical parameters over the course of LVAD support.

*In silico* analysis failed to show genes shared by the four DEmiRs whereas, after excluding miR-451a, the other three miRs have 13 common target genes, mainly involved in metabolic and oxygen-related cell responses (Supplementary File S1, Figure S2). Of the targeted signaling pathways shared by the four DEmiRs, 36 were of potential relevance in cardiac pathogenesis (Supplementary File S2).

No significant longitudinal changes were found in PPP for all the analyzed miRs.

### 3.4. Platelet miRs expression and HRAEs

The low number of thromboembolic events (*n *= 1) hampered a formal correlation analysis of platelet miRs expression and this category of HRAEs. Conversely, the expression levels of platelet miRs were compared in patients who did suffer from bleeding events (*n *= 5) vs. those who did not develop any HRAE (*n *= 9).

**Figure 5:**
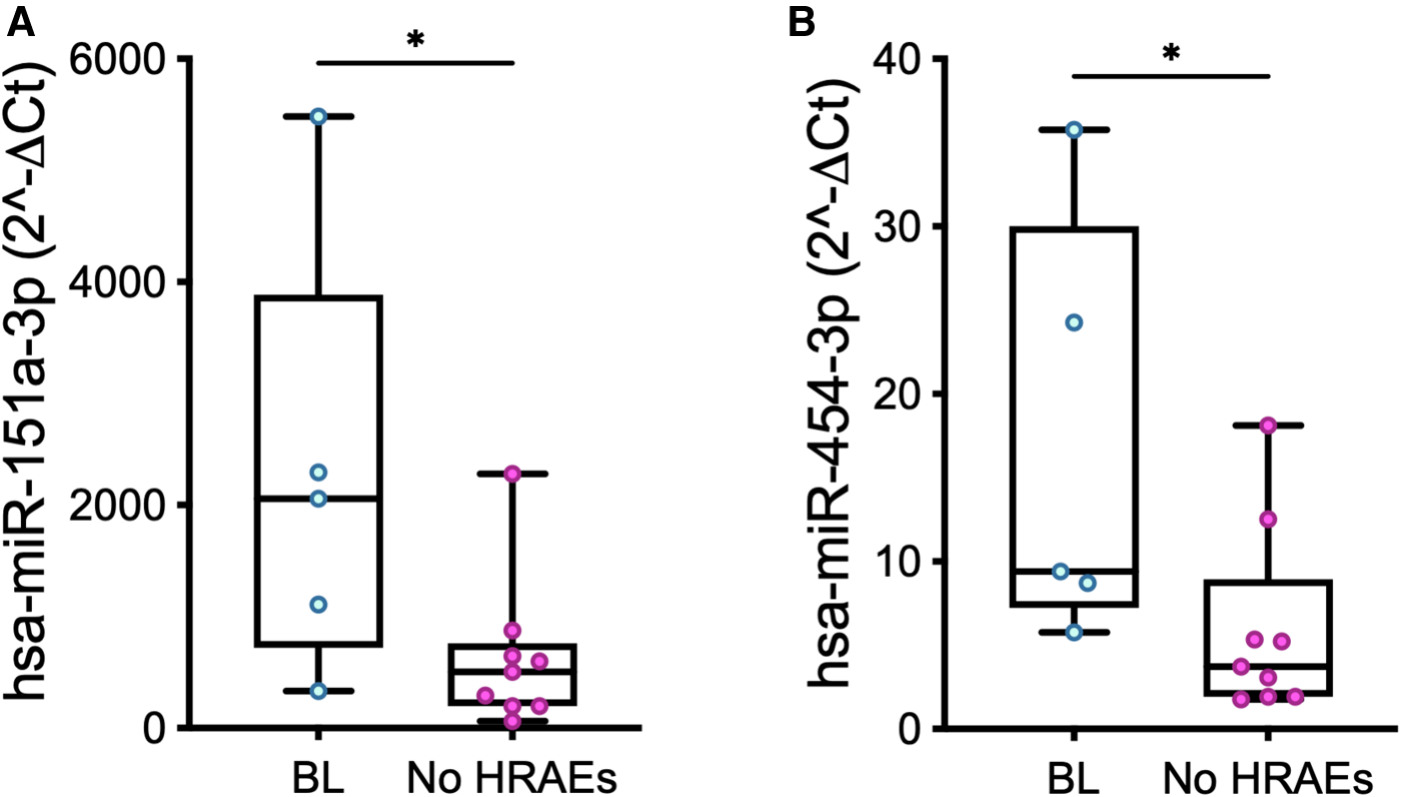
Baseline (t0) expression levels of miR-151a (**A**) and miR-454 (**B**) measured in PRP in patients who developed a bleeding event over the course of LVAD support (BL, *n *= 5) vs. those who did not suffer from HRAEs (no HRAEs, *n *= 10). Values are presented as boxes: dots indicate single values; whisker bars indicate min and max. *p < 0.05.

Pre-implant expression levels of miR-151a and mir-454 in PRP were higher in patients who suffered from a bleeding event (Figure 5; miR-151a: *p *= 0.04; miR-454: *p *= 0.03).

*In silico* analysis showed different pathways involved in cardiac physiopathology targeted by miR-151a (*n *= 79) or miR-454 (*n *= 108), including one associated with the complement cascade; some of them (*n *= 58) appeared shared by both miRs (Supplementary File S3).

**Figure 6:**
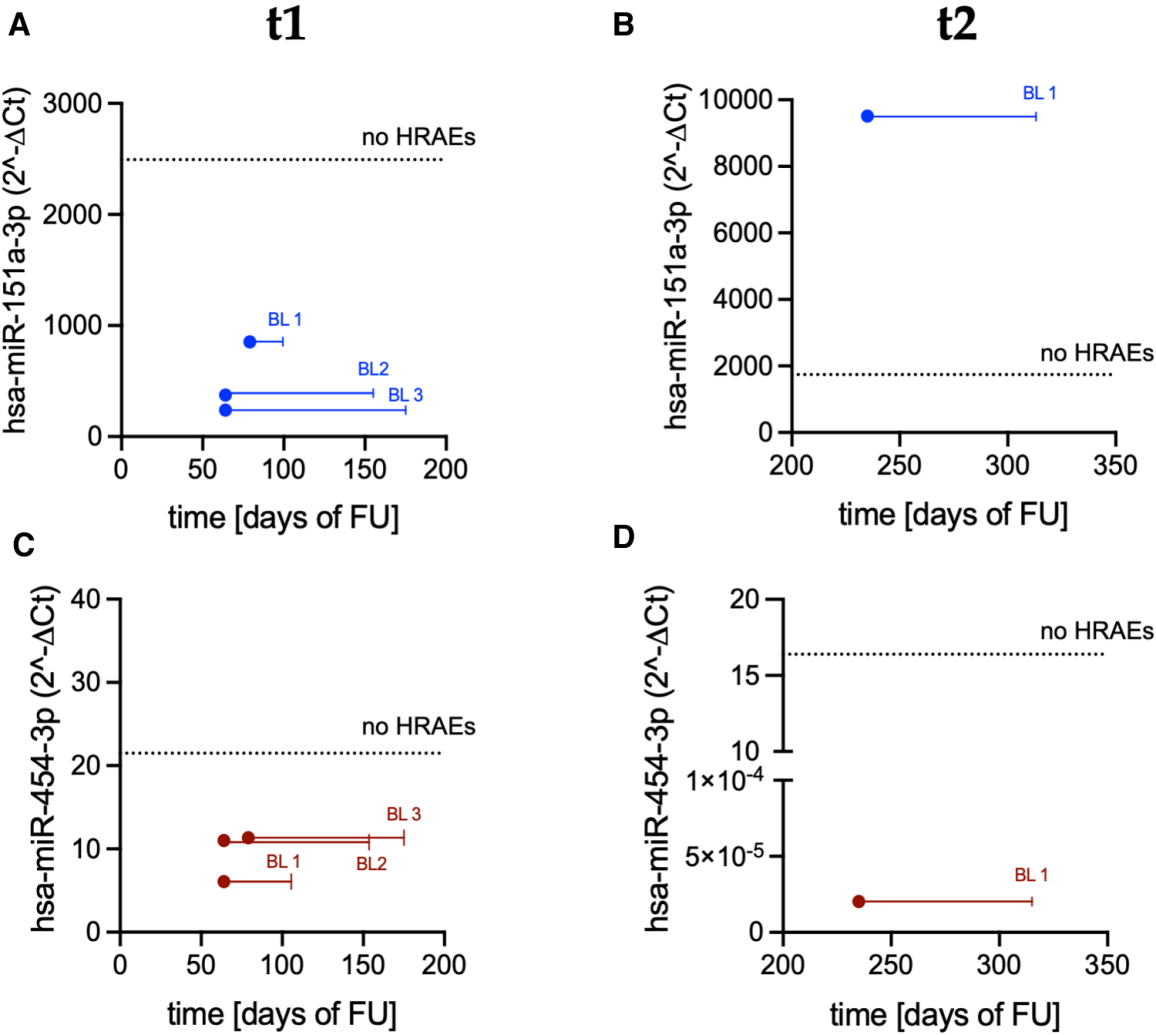
Expression levels of miR-151a and miR-454 measured in PRP before the occurrence of a bleeding event: (**A**) miR-151a at t1; (**B**) miR-151a at t2; (**C**) miR-454 at t1; and (**D**) miR-454 at t2. Dots indicate miRs expression level in bleeders (BL; t1: *n *= 3; t2: *n *= 1). Solid lines indicate temporal distance from the miR measurements to the occurrence of the bleeding event [t1: measurements performed 91 days (median value) before the occurrence of the events; t2: measurements performed 80 days before the occurrence of the event]. Dashed lines indicate the median value of miRs expression in patients who did not suffer from HRAEs (no HRAEs; t1: *n *= 8; t2: *n *= 7).

An altered expression of the same miRs was also recorded in bleeders early before the occurrence of a bleeding event (Figure 6). Specifically, the median values of miR-151a were 7.8-fold lower at t1 (Figure 6A; *p *= 0.03) and 5.46-fold higher at t2 (Figure 6B) in bleeders *vs.* patients who did not suffer from HRAEs; the median values of miR-454 were 1.94-fold and >800-fold lower at t1 (Figure 6C) and t2 (Figure 6D), respectively, in bleeders *vs.* HRAEs-free patients.

The miRs expression levels at t3 were not analyzed as no data anticipating the events were available (i.e., the events occurred before the scheduled time-point for blood sampling and miR analysis).

## 4. Discussion

In this proof-of-concept study, we evaluated, for the first time, the expression levels of platelet miRs in the setting of durable LVAD support. Previous works analyzed circulating/tissue miRs in LVAD patients (18–22), but whether changes in platelet miR patterns occur after LVAD implantation remained unexplored.

Our preliminary results suggest a modulatory effect of LVADs upon the platelet miRs processing machinery: this is consistent with the longitudinal changes in the expression levels of four platelet miRs found in PRP over the course of LVAD support (Figure 3).

### 4.1. Pre-implant DEmiRs: the role of HF and temporary mechanical circulatory support

Baseline miRs differences in patients vs. controls (Figure 1) are likely due to the pathophysiology of advanced HF and targeted medical therapy and are in agreement with previous studies on platelet miRs expression in HF (29–35). *In silico* analysis highlights a common target gene shared by the four DEmiRs, i.e., the SCN1A, which is known to encode for a tetrodotoxin-sensitive cardiac voltage-gated sodium channel (36). Whether SCN1A is a possible pathogenic player in advanced HF and might have any functional role during LVAD support deserves further dedicated investigation.

Our data also confirm good specificity of miRs to differentiate HF patients according to clinical characteristics (Figures 2A–C) (37–39) and provide preliminary evidence of a distinct platelet miR pattern correlated with pre-implant temporary mechanical circulatory support devices (Figure 2D). These observations, together with their correlation with the clinical course of the patients, deserve further investigation since they may have strong implications for clinical practice, namely, to guide patient-tailored management strategies. Indeed, we can argue that hemodynamic phenomena characteristics of the Impella device (e.g., cardiac unloading, shear stress, blood flow patterns) may have a contributory role in modulating platelet miRs expression, in addition to HF itself.

### 4.2. Changes in platelet miRs expression driven by LVAD support: recovery of HF symptoms, platelet activation, and structural changes of the supported heart

Following LVAD implantation, interestingly, we observed that pre-implant differences in patients vs. controls did not normalize, i.e., values in patients did not recover to the median control values over the course of LVAD support (Figure 3), suggesting that LVADs prompt a distinctive, LVAD-specific, and miR-related platelet “phenotype.”

These data indicate that after LVAD implantation, the reverse of miRs expression do not match the reverse of signs and symptoms of HF (Table 3) and suggest that platelet miRs may respond to a complex pattern of stimuli beyond HF symptoms. These might include systemic alterations common to LVAD patients (changes in macro- and micro-circulation or coagulation patterns) as well as the effect of medical therapy and mechanical stimuli (shear forces) provided by the device. Interestingly, no differences were observed in the expression levels of platelet miRs between patients implanted with the HVAD or the HM3 (data not shown).

According to previous works on platelet miRs expression, we speculate that our data mirror platelet function abnormalities largely described in LVAD patients [chronic activation/hyper-reactive status, and degranulation and impaired adhesion and aggregation capability (4, 8, 9, 40–43)]. In detail, the progressive increase in the expression levels of platelet miR-25 and miR-451a from t0 to t3 (Figures 3A,B) might indicate an increase in platelet activation over the course of LVAD support (44). Conversely, the changes in the expression of miR-320a and miR-144, two recognized regulators of platelet reactivity, activation, signaling, and aggregation, as well as degranulation (45), did not delineate a pattern over time (Figures 3C,D), which might reflect partial and/or transient activation of platelets and deserve further investigation. We also hypothesize that lack of a clear pattern of expression over time might reflect a continuous “adaptation” of platelet miRs expression to the continuously evolving clinical features of the patients: as discussed earlier, platelet miRs appear to respond indeed to a complex and heterogenous pattern of stimuli. Studies evaluating further changes in platelet miRs expression over longer periods of LVAD support might contribute to clarify these phenomena.

The four DEmiRs shared molecular pathways including that of peroxisome proliferator activated receptor (PPAR), a regulator of platelet activation (46) and TNF-α and mammalian target of rapamycin (mTOR), which have been found to be involved in bleeding (47–50).

Our hypothesis of a dynamic re-programming of platelet miRs toward a low pro-thrombotic (rather pro-hemorrhagic) state over the course of support is corroborated by the correlation between platelet miR-126 and D-Dimer and AT-III plasma levels: indeed, the observed negative (D-Dimer, Figure 4A) and positive (AT-III, Figure 4B) relations with miR-126 might be compatible with lack of pro-thrombotic activation and retention of mature miR-126 into platelets (51). In this regard, it is worth noting that only one patient in our cohort developed a thromboembolic complication, while five (33%) suffered from bleeding. Nevertheless, whether the increase of miR content in platelets (retention) effectively reflects low level of activation or preludes the release of pro-thrombotic miRs needs to be clarified in dedicated mechanistic studies.

According to described mechanisms of genomic, molecular, cellular, and structural changes of the LVAD-unloaded left ventricle (52), we also examined possible correlations between the changes in platelet miRs expression and changes in cardiac function driven by LVAD support. *In silico* analysis showed different signaling pathways shared by the four DEmiRs having potential relevance in cardiac pathogenesis (Supplementary File S3). Furthermore, the positive correlation found at t3 between platelet miR-126 and LVEF (Figure 4C) confirms the prognostic role of this miR in cardiovascular disease (53), and further supports its association with cardiac function.

### 4.3. Changes in platelet miRs expression and adverse events

Our study also suggests that abnormal expression levels of platelet miRs might represent novel biomarkers predictive of bleeding events in the LVAD population.

To date, prognostic biomarkers of bleeding relevant to platelet function abnormalities in LVAD patients are scarce. Previous studies demonstrated that acquired platelet dysfunction, secretion defects, and impaired aggregation capability contribute to the development of bleeding in LVAD patients (9, 54–57). Recently, reduced pre-implant expression of platelet P-selectin and GPIIb/IIIa has been proposed to predict bleeding while on LVAD (58).

The pre-implant expression levels of platelet miRs-151a and miR-454 allowed to stratify the subgroup of patients who suffered from bleeding with respect to patients who did not develop HRAEs (Figure 5). Indeed, we reasoned that thromboembolic and bleeding events might be associated with different miR patterns: accordingly, to avoid bias and enhance selective focus on bleeding events, data of the patient who suffered from stroke were excluded.

Our data suggest the existence of a platelet miR-related fingerprint of bleeders, i.e., the retention of miR-151a—classified as thrombomiR—and miR-454—known to be involved in the regulation of the complement cascade (59, 60). On the other hand, the potential targets of these miRs (inside vs*.* outside platelets) were not identified. In other words, we did not investigate whether a change in the levels of expression of these miRs effectively influences platelet function or activation or whether platelets are only acting as carriers for the miR. Accordingly, we are not proposing altered levels of expression of miRs by platelets to be at the origin of the bleeding event, rather suggesting that the platelet miRs represent novel markers of a pathogenic state (increased risk of bleeding) in some patients. Future mechanistic studies on the correlation between the expression levels of platelet miRs and traditional markers of platelet function/activation (e.g., P-selectin; CD40l and/or PF4), and on the mutual contribution of altered miR patterns and other recognized risk factors to the development of HRAEs are needed to elucidate these phenomena and provide further insights into pathophysiology of LVAD-related complications. As far as potential age-related inferences on altered expression levels of platelet miRs in bleeders is concerned, of note, age at implant was not statistically different in the two groups (bleeders vs. non-HRAEs patients).

On the other hand, no significant differences were found in the expression levels of platelet miR-151-a and miR-454 between bleeders and controls (*p *= 0.56 and *p *= 0.75, respectively): these data highlight the need for further analyses aimed at validating a possible contributory role of platelet miRs profile to predispose LVAD patients to HRAEs.

Differences in the expression levels of miR-151a and miR-451 were also observed in bleeders early before the clinical manifestation of the event (Figure 6), further corroborating their prognostic power and the clinical utility of monitoring platelet miRs in LVAD patients. Conversely, coagulation parameters were comparable in bleeders vs. HRAEs-free patients (data not shown) and did not warn for an increased risk of bleeding.

Downregulation of platelet miR-151a in bleeders at t1 possibly indicates abundance of hypo-reactive platelets with inhibited aggregation capability (61, 62). However, the patient who suffered from bleeding at t2 showed an upregulation of mir-151a levels anticipating the event (Figure 6B). This apparent discrepancy might be explained by the different types of bleeding (Table 3) or a modulatory effect of systemic infection, as driveline infection was concomitant in 3 out of the 7 bleeding events (43%). Moreover, we cannot exclude a contribution by other factors (e.g., adverse events not related to hemocompatibility or peculiar conditions and management strategies at the time of the event)—alone or in concert—to different miR-151a expression. In this regard, we emphasize that durable LVAD support is (intrinsically) a multisystemic and dynamic scenario, where features that might amplify the risk of HRAEs and impact platelet miRs expression might be present at baseline or emerge later after LVAD implantation.

## 5. Study limitations

This is a single-center observational study performed in a small cohort of patients. Potential gender-related differences in platelet miRs expressions were not evaluated. Future studies on a larger, multi-center cohort are therefore warranted to increase the power and significance of our results. In particular, studies on a larger number of patients are required to validate differences in miR patterns associated with different devices. Furthermore, the cut-off miRs expression levels that are predictive of HRAEs were not identified. Moreover, the functional role and clinical implications of some DEmiRs in the specific scenario of LVAD support were not fully elucidated, nor the associated downstream signaling pathways validated. Also, we did not investigate possible association and/or cross-talks between altered miRs expression and different pro-thrombotic/pro-hemorrhagic risk factors in the development of HRAEs, including acquired von Willebrand syndrome and platelet activation levels, nor inferences of changes in anticoagulation/antiplatelet therapy over the course of support.

## 6. Conclusions

We provide a proof-of-concept evidence of significant modulation of platelet miRs expression driven by durable LVADs and suggest a potential of platelet miRs signature to identify patients at high risk for HRAEs, specifically bleeding. Indeed, we propose two miRs candidates, miR-151a and miR-454, deserving further evaluation for future clinical use. Thus, our study might have important translational relevance related to novel strategies for the prevention of HRAEs targeted to platelet miRs expression, which might overcome evident limitations of available coagulation markers, prediction algorithms, and point-of-care coagulation tests. Future studies to validate our preliminary findings are warranted.

## Data Availability

The raw data supporting the conclusions of this article will be made available by the corresponding authors upon reasonable request.
