# Supplementary material for: Left ventricular assist devices promote changes in the expression levels of platelet microRNAs
Source: Front Cardiovasc Med. 2023 Jun 15;10:1178556. doi: 10.3389/fcvm.2023.1178556 (PMC10308775; doi:10.3389/fcvm.2023.1178556)
Supplement: Supplementary file 1 [file Table1.docx]

Supplementary Material

Left ventricular assist devices promote changes in the expression levels of platelet micro-RNAs

**Maria Lombardi, Marta Bonora, Luca Baldetti, Marina Pieri, Anna Mara Scandroglio, Giovanni Landoni, Alberto Zangrillo, Chiara Foglieni*, Filippo Consolo2*.**

***Correspondence:**

Filippo Consolo: [consolo.filippo@unisr.it](mailto:consolo.filippo@unisr.it)

and

Chiara Foglieni: [foglieni.chiara@hsr.it](mailto:foglieni.chiara@hsr.it)

# Supplementary Methods

**1.1 Blood processing and microRNA measurement**

Blood samples for miRs were obtained from patients and controls via venipuncture or central line (venous or arterial catheter) and collected in 5mL EDTA tubes (BD Vacutainer®). The samples were processed within 15 minutes from the collection under sterile conditions to obtain platelet-rich plasma pellets (PRP) and platelet-poor plasma (PPP) [1], both stored at -80°C until the time of the analysis. Repeated freeze-thaw cycles were avoided to ensure detection [2]. Blood samples showing preanalytical hemolysis were discarded.

The miR-enriched fraction was extracted from PRP and PPP using miRNeasy mini kit and miRNeasy serum/plasma miR isolation kit (QIAGEN S.r.l., Italy), respectively. Total RNA was quantified at NanoDrop™ 1000 (Thermo Fisher Scientific, Inc., Waltham, MA, US). Synthesis of cDNA was carried out by TaqMan® Advanced miRNA Assay (Invitrogen, Carlsbad, CA, USA). Spectrophotometric measurements performed at NanoDrop™ 1000 excluded the presence of relevant contaminating hemolysis (*i.e.,* absence of relevant peaks at 414-nm wavelength [3]).

RT-qPCR was carried out by Taq-Man® Fast Advanced Master Mix with Taqman primer/Fam-labeled probes (Applied Biosystems, Foster City, CA, USA) to determine the selected miRs. Samples were run in triplicate, and miR levels were normalized to that of miR-103a-3p (PRP) or miR-16-5p (PPP) [4]. The miR expression levels in PRP were also normalized against platelet count in the blood sample. Relative expression was determined using the ΔCt method. All the procedures were carried out according to manufacturers’ instructions.

In silico analysis aimed at identifying pathways targeted by differentially expressed miRs (DEmiRs) was performed using the miRnalyze online tool [5].

**1.2 References**

1. Dhurat, R., Sukesh, M. (2014) Principles and Methods of Preparation of Platelet-Rich Plasma: A Review and Author's Perspective. J Cutan Aesthet Surg 7(4):189-97.
2. Muth, D.C., et al. (2018) miRNAs in platelet-poor blood plasma and purified RNA are highly stable: a confirmatory study. BMC Res Notes 11(1):273.
3. Kirschner, M.B. et al. (2011) Haemolysis during sample preparation alters microRNA content of plasma. PLoS One 6(9):e24145.
4. Donati, S. et al. (2019) Human Circulating miRNAs Real-time qRT-PCR-based Analysis: An Overview of Endogenous Reference Genes Used for Data Normalization. Int J Mol Sci 20(18):4353.
5. Subhra Das. S. et al. (2017) miRnalyze: an interactive database linking tool to unlock intuitive microRNA regulation of cell signaling pathways. Database (Oxford) 2017(1):bax015.

# Supplementary Figures and Tables

**Supplementary Figure 1:** Comparison of miRs expression levels in PRP and PPP between LVAD patients at baseline (t0) and healthy volunteers (controls, CTRL). The miRs that were not differently expressed in the two groups are shown (*p*>0.05): **(a)** miR-19b **(b)** miR-25 **(c)** miR-144 **(d)** miR-151a **(e)** miR-382 **(f)** miR-454. Values are presented as boxes: dots indicate single values; whisker bars indicate min and max.

**
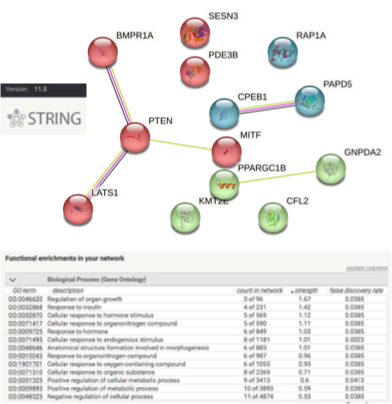
**

**Supplementary Figure 2:** Analysis of the putative network interactions among the proteins encoded by genes targeted by miR-25, miR-320a and miR-144 obtained with STRING v11.5 online database. Gene ontology annotation pathways relating to these proteins are listed.

| **Supplementary Table 1.** A synopsis of selected published data related to platelet miRs investigated in this study. | |
| --- | --- |
| **Expression of Platelet miRs** | **Reference DOI** |
| - top 20 platelet miRS - abundance: miR-223, miR-126, miR-16, miR-19b, miR-151 | 10.3109/09537104.2011.560305 |
| - top 40 platelet miRs: miR-223, miR-126, miR-320, miR-451a, miR-151-5p, miR-20b, miR-374b, miR-19b ranked by expression | 10.5772/intechopen.93181 |
| - top level platelet miRs: miR-223, miR-16; top DEmiRs: miR-320a, miR-144 | 10.1182/blood-2010-09-299719 |
| - profiling of 377 platelet miRs: top DEmiR: miR-223 in PRP vs. PPP | 10.1161/CIRCRESAHA.111.300539 |
| - difference in classification top 10 platelet miR expression levels by different studies_ includes miR-126, miR-223, miR-320a, miR-151amiR-19b, miR-25 | 10.1161/CIRCRESAHA.116.309303 |
| - miR-454 🡻 in reticulated vs. mature platelets | 10.1055/s-0039-1695009 |
| - **Platelet miRs and platelet activation** |  |
| - 🡻 platelet miR-223 in association with 🡹 in platelet reactivity, despite treatment with clopidogrel | 10.3109/09537104.2011.560305 |
| - platelet activation in T2DM 🡪 transfer of miR-126, miR-223, miR-16, and miR-423 from PRP to PPP, rescued with the addition of aspirin | 10.1093/eurheartj/eht007 |
| - miRs involved in platelet activation: miR-223 (P2Y12), miR-126 angio-miR | 10.3109/09537104.2012.724483 |
| - reprogramming of the platelet miRNome during activation involving in vitro miR-19b, miR-382, miR-103, miR-151a, miR-374b, miR-20b, miR-144, miR-126, miR-454 | 10.1160/TH14-09-0726 |
| - release of thrombomiRs by activated platelets in PRP and comparison with PPP content: abundance of miR-223, miR-320a, miR-126 + miR-151a | 10.3390/cells11081254 |
| - relationship between miR-223 in platelet reactivity and antiplatelet therapy | 10.1155/2015/981841 |
| **Platelet miRs and cardiovascular diseases** |  |
| - platelet miR-144, miR-223, miR-451, miR-454: changes in thrombotic states | 10.1155/2016/2872507 |
| - TF-targeting miRs: miR-19 in hemostasis, miR-223 in CVD; modulator of endothelial cells thrombogenicity: miR-126; miR-126 changes are driven by inflammatory TNF-α | 10.1055/s-0037-1606568  10.1161/ATVBAHA.115.306 |
| - 🡻 miR-223 levels in hypertension vs. healthy subjects, 🡻 miR-223 and miR-126 in CVD | 10.1038/s41371-018-0123-5 |
| - platelet miR-126 and miR-223 involvement in AMI | 10.1007/s11239-017-1537-6  10.1016/j.jacc.2012.03.056 |
| - changes in platelet miRs, including miR-454: association with premature CAD | 10.1371/journal.pone.0025946 |
| **miRs and LVAD support** |  |
| - DEmiRs in LVAD cardiac tissues: miR-374b, miR-144, miR-223 | 10.1155/2015/592512 |
| - miR-126 and miR 320 in LVAD patient plasma samples | 10.1161/circ.130.suppl_2.15556 |
| - 🡹 circulating miR-126 at follow up visits after LVAD implantation | 10.1016/j.ijcard.2021.06.050 |
| - in cardiac tissue: 🡻 miR-144 but no changes in miR-25 in patients pre-LVAD vs. post-LVAD; no differences in miR-144, miR-451, 320a in patients pre-LVAD vs. controls | 10.1371/journal.pone.0136404 |
| miRs: microRNAs; DEmiRs: differentially expressed miRs; PRP: platelet-rich plasma; PPP: platelet-poor plasma; T2DM: type 2 diabetes mellitus; TF: tissue factor; CVD: cardiovascular diseases; TNF-α: tumor necrosis factor α; AMI: acute myocardial infarction; CAD: coronary artery disease; LVAD: left ventricular assist device. | |

**
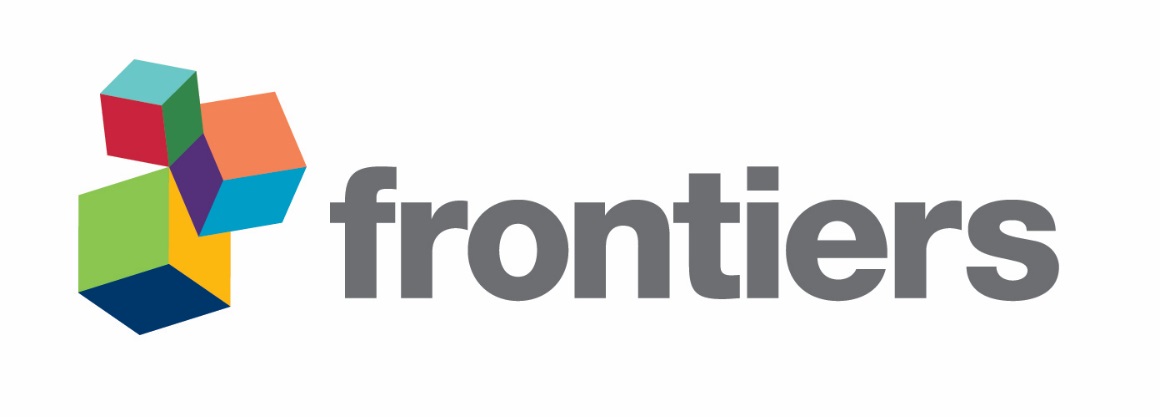
**
